# Isotemporal Substitution Analysis of Accelerometer-Derived Sedentary Behavior and Physical Activity on Cardiometabolic Health in Korean Adults: A Population-Based Cross-Sectional Study

**DOI:** 10.3390/ijerph182111102

**Published:** 2021-10-22

**Authors:** Jiameng Ma, Hyunshik Kim, Junghoon Kim

**Affiliations:** 1 Faculty of Physical Education, Sendai University, Miyagi 989-1693, Japan; jm-ma@sendai-u.ac.jp (J.M.); hs-kim@sendai-u.ac.jp (H.K.); 2 Sports and Exercise Medicine Laboratory, Korea Maritime and Ocean University, 727 Taejong-ro, Yeongdo-gu, Busan 49112, Korea

**Keywords:** sedentary behavior, physical activity, accelerometer, cardiometabolic health, KNHANES

## Abstract

Reducing sedentary behavior and increasing physical activity may be important for maintaining good cardiometabolic health. However, many studies have focused on the independent effect of sedentary behavior and physical activity, but it is unclear whether replacing time spent in sedentary behavior with physical activity is beneficial for cardiometabolic health. Therefore, this population-based cross-sectional study aimed to investigate the effect of behavioral transformations between sedentary behavior and level of physical activity on cardiometabolic health in Korean adults using data from the Korea National Health and Nutrition Examination Survey (KNHANES) 2014–2017. The study participants included 2197 adults from the KNHANES. In the partition model, moderate-to-vigorous physical activity (MVPA) was significantly associated with adverse cardiometabolic health, adjusted for potential confounding factors. The odds ratio for adverse cardiometabolic health significantly decreased with the replacement of sedentary behavior and light intensity activity with MVPA in the isotemporal substitution model (*p* < 0.05). In the models stratified by sex, we observed significant associations between handgrip strength and cardiometabolic health in women (*p* < 0.001), but not in men. Thus, our findings suggest that replacing sedentary behavior with MVPA may contribute to improved cardiometabolic health.

## 1. Introduction

Cardiometabolic diseases are a public health problem worldwide [1]. It represents a leading cause of disease burden and includes metabolic syndrome, ischemic heart disease, diabetes, and stroke [1]. As the prevalence of obesity and cardiometabolic disorders has increased worldwide, including in South Korea [2,3,4,5], various studies are being conducted to examine these issues. Cardiometabolic disease is primarily associated with an increase in sedentary behaviors [6,7,8].

The World Health Organization (WHO) published the “WHO 2020 guidelines on physical activity and sedentary behavior.” These guidelines aim to reduce physical activity deficits in all age groups as a Global Action Plan goal. The WHO is working globally to reduce individuals’ sedentary hours and increase their physical activity [9]. Sedentary behavior is defined as any waking behavior characterized by an energy expenditure of ≤1.5 metabolic equivalents, while sitting, leaning, or lying [10]. Epidemiological evidence suggests that habitual sedentary behavior is associated with an increased risk of obesity, metabolic syndrome, cardiovascular disease, and premature mortality [11,12,13,14,15]. It is also important to note that even if the daily physical activity guidelines are followed, prolonged sedentary behavior is an independent risk factor associated with cardiovascular risk factors [16].

Various human behaviors are interdependent. For example, the field of motor dynamics depicts daily activities during waking hours to be composed of sedentary behavior, light-intensity activity, and moderate-to-vigorous physical activity (MVPA). This is because there is a limited amount of time in a day at a person’s disposal, and individuals must reduce the time spent on one activity to devote some to others. For example, to increase the time spent on moderate to vigorous exercise by 30 min, the time spent on another activity needs to be decreased by that much. This indicates that the periods of time spent on different actions in a day share an interdependent relationship. Willett et al. [16] proposed that total energy intake should be considered when investigating the relationship between nutritional intake, especially that of major nutrients, and disease. The isotemporal substitution (IS) analysis model is defined as “a method of estimating the effect on an objective variable when one action is replaced by an equivalent amount of another action”. The IS model has been reported as an isocaloric replacement analysis model similar to the proposed analytical models and is characterized by an interpretation that is closer to people’s real life than that offered by conventional analytical models because it considers the interdependence of behaviors [17]. In exercise epidemiology, previous studies have reported that reallocating 30 min/day of sedentary time with light activity reduced mortality risk by 20% over a 5-year follow-up period [18]. In addition, substituting light activity for vigorous physical activity affected indicators of obesity, such as weight and body fat mass [19]. Female adults’ weight was reduced by 1.57 kg when 30 min of walking was substituted by jogging during the day; moreover, when 30 min of watching TV was substituted by walking during the day, their weight was reduced by 1.14 kg [20].

Previous studies in Korean adults have focused on health problems related to the amount of physical activity and sedentary behavior. However, the interdependent relationship between the two is unclear. Studies that examine the relationship between sitting behavior and the level of physical activity by applying the IS model to cardiometabolic disease are limited. According to the results of the Korea National Health and Nutrition Survey (KNHANES), for the past decade, the prevalence of obesity-related metabolic syndrome in Korean adults was reported to be 22.4% [21]. Therefore, evaluating the transformation of each physical activity may be significant. Moreover, if the results offer specific suggestions and a strategy to reduce sedentary behavior, a complex population approach that is practical and beneficial could be established.

Therefore, this population-based cross-sectional study aimed to investigate the effect of behavioral transformations between sitting time and level of physical activity on cardiometabolic disease in Korean adults. We hypothesized that isotemporal substitution of sedentary behavior with any type of activity contributes to a decreased risk of cardiometabolic disease.

## 2. Materials and Methods

### 2.1. Study Participants

For this study, we used data from KNHANES 2014–2017. KNHANES is a series of cross-sectional surveys of nationally representative samples of a civilian, non-institutionalized population conducted to assess the health and nutritional status of the Korean population [22]. The KNHANES surveys were approved by the Institutional Review Board (IRB) of the Korea Centers for Disease Control and Prevention (KCDC), and all participants provided written informed consent (IRB number: 2013-12EXP-03-5C).

Physical activity measurements using accelerometers were conducted in KNHANES 2014–2017 for adults aged 19–64 years in 2014–2016 and for those aged ≥65 years in 2017. The process of accelerometer measurement was explained to the participants who participated in the health interview during KNHANES (excluding those who had disability), and the accelerometers were distributed.

A total of 2927 adults agreed to participate in the accelerometer measurements. Participants (*n* = 110) who lost their accelerometer, those who did not wear it, those who experienced malfunction with it, and those had data errors with the accelerometer were excluded (adherence: 96.2%). Next, from a total of 2817 participants, we excluded 65 participants who had missing data regarding fasting blood samples and eight due to sociodemographic and lifestyle characteristics. We then excluded 547 participants who did not ware an accelerometer for ≥4 days for ≥10 h a day. Finally, 2197 participants were included in the study.

### 2.2. Accelerometer Measures of Physical Activity and Sedentary Behaviors

Sedentary time and each physical activity type were measured using a triaxial accelerometer (ActiGraph GT3X, LLC, Fort Walton Beach, FL, USA) (Figure S1). The accelerometer was worn on the left (or right) side of the waist based on the navel for 7 consecutive days from the day consent was obtained. The accelerometer was set to record from midnight (12 a.m.) the day after consent was obtained, and it was connected to a customized elastic belt for participants’ comfort. In addition, they were instructed to wear it for all activities related to school or work, but not when swimming or showering.

The frequency of summarizing the electrical signals was set to 1-min epochs in consideration of the subject’s physical activity characteristics and the storage capacity of the accelerometer [15,23]. A continuous zero count of at least 1 min was defined as the non-wear time. We calculated the wear time by subtracting the non-wear time (only if the non-wear time was continued for >60 consecutive min). We also considered invalid records if they did not have at least 10 h of wear time per day for four days [24].

Sedentary behavior was defined using a threshold of <100 counts/min and light intensity defined using a threshold of <760 counts/min. Lifestyle activity was defined using a threshold of <2020 counts/min, and MVPA was defined using a threshold of ≥2020 counts/min. We computed the mean value per valid day from accelerometer records to calculate the time spent in sedentary behavior, light and lifestyle activities, and MVPA [25]. The procedure used in this study has been described in the physical activity monitoring protocol and our previously published research [15,23,25,26].

### 2.3. Blood Sample Test

Blood samples were obtained from all participants after >8 h of overnight fasting. All blood samples were analyzed using a COBAS 8000 C702 (Roche, Mannheim, Germany). Fasting glucose levels and hemoglobin A1c (HbA1c, %) were analyzed using the hexokinase method, and triglycerides, high-density lipoprotein (HDL in mg/dL) cholesterol (mg/dL), and total cholesterol (TC in mg/dL) were analyzed by implementing the enzyme method (Hitachi, Tokyo, Japan).

### 2.4. Cardiometabolic Risk Factors

We considered the following cardiometabolic risk factors: waist circumference (WC), systolic (SBP) and diastolic blood pressure (DBP), fasting glucose, HbA1c, triglycerides, HDL-cholesterol, and TC. In addition, we developed binary variables for each cardiometabolic risk factor based on previous studies among Korean adults. Abdominal obesity was defined as WC ≥90 cm for men and ≥80 cm for women. High blood pressure was defined as SBP ≥130 mmHg and/or DBP ≥85 mmHg. High glucose was defined as a fasting (>8 h) glucose level of ≥100 mg/dL and high HbA1c ≥6.5%. High TC level was defined as ≥190 mg/dL. Low HDL cholesterol was defined as <40 mg/dL for men and <50 mg/dL for women. Finally, we also computed the total number of adverse cardiometabolic risk factors and defined ‘high risk’ as ≥3 adverse or ≥4 adverse cardiometabolic risk factors [27,28].

### 2.5. Covariates

Several variables, such as sociodemographic factors (age, sex, education level, and household income), smoking status, and alcohol consumption, were used as confounding factors. Educational level was categorized into three groups: <high school, high school, and >high school. Household income was categorized into four groups based on quartiles. Smoking status was categorized as never, former, or current. Alcohol consumption was categorized as never, once a week, 2–4 times/week, and ≥4 times/week. Body mass index (BMI) was calculated using the following formula: body weight/height (kg/m^2^).

### 2.6. Statistical Analysis

All analyses were conducted using the SAS ver. 9.4 (SAS Institute, Cary, NC, USA), and statistical significance was set at *p* < 0.05. The study participants’ characteristics, including demographics and proportion of cardiometabolic risk factors, were presented as percentages (%), and statistical significance was analyzed using the chi-square test. We estimated the mean and standard error (SE) for continuous variables (cardiometabolic risk and level of physical activity) by sex using independent *t*-tests.

We developed three different logistic regression models for each type of activity on the risk of adverse cardiometabolic health (≥3 or ≥4 risk factors) to assess the effects of sedentary behavior. We calculated the odds ratio (OR) and confidence interval (CI) for adverse cardiometabolic risk according to the change in activity time every 30 min.

First, we developed single-variable models to evaluate individual associations of sedentary behavior, light intensity, lifestyle activities, and MVPA (including only one type of activity) after adjusting for demographic factors, including age, sex, education level, household income, alcohol consumption, smoking status, and total accelerometer wear time.

Second, we developed partition models to examine independent associations for each type of activity, wherein sedentary behavior and all types of activities (times spent during light intensity activity, lifestyle activity, and MVPA), and covariates were applied to one model without the total accelerometer wear time.

Finally, the IS models were used to estimate the association of replacing 30 min/day of time spent on one activity with the equal time spent on other activities and the risk of adverse cardiometabolic health with total accelerometer wear time. For example, to evaluate the effects of replacing 30 min of sedentary behavior with light, lifestyle, and MVPA, sedentary time was dropped from the full model, including other types of activities and total wear time after adjusting for covariates. In this case, the OR of MVPA represents the effect of a 30 min/day substitution of sedentary time with MVPA while keeping the other activity variables and total wear time constant.

In addition, we performed a sensitivity analysis using models stratified by sex (men or women) and age group (<65 years or ≥65 years) to evaluate sex and age-related differences in these associations.

## 3. Results

Table 1 shows the overall characteristics of the participants according to sex. The mean age was 48.92 ± 0.33 years, and the mean BMI was 23.80 ± 0.07 kg/m^2^. The mean WC, SBP, and DBP were 80.95 ± 0.21 cm, 116.72 ± 0.35 mg/dL, and 74.60 ± 0.21 mg/dL, respectively. The mean total number of cardiometabolic risk factors was 2.33 ± 0.05 in men and 1.94 ± 0.04 in women (*p* < 0.001).

Time spent of sedentary time, light intensity and lifestyle activities, and MVPA were 465.45 ± 3.47 min/day, 216.23 ± 1.95 min/day and 79.42 ± 1.18 min/day, and 32.63 ± 0.73 min/day, respectively. The total wear time of the accelerometer was 793.74 ± 4.63 min/day. Light intensity activity was significantly longer in the female population than in the male population. However, the time spent on MVPA was significantly longer in men than in women (*p* < 0.001).

Table 2 presents the proportion of individual cardiometabolic risk factors and adverse cardiometabolic health (≥3 or ≥4 risk factors) according to sex. The proportions of abdominal obesity, high blood pressure, high fasting glucose, high TG, and high TC were significantly higher in men than in women (Table 2). Furthermore, the percentage of adverse cardiometabolic health (≥3 risk factors) was 45.04% in men and 32.46% in women (Table 2, *p* < 0.001).

Table 3 shows the relationship between sedentary behavior and each type of physical activity with adverse cardiometabolic health (≥3 risk factors) in the single, partition, and IS models. In the single-factor models, we found a significant association in MVPA (OR: 0.89, 95%CI: 0.81–0.97) after adjusting for covariates. In the partition models, MVPA was negatively associated with adverse cardiometabolic health (OR: 0.87, 95%CI: 0.80–0.96). The following addresses the changes in the risk of adverse cardiometabolic health according to the substitution of each type of activity for the others. In the IS models, replacing sedentary behavior (OR: 0.88, 95%CI: 0.80–0.97), light intensity activity (OR: 0.87, 95%CI: 0.79–0.96), and lifestyle activity (OR: 0.85, 95%CI: 0.75–0.97) with 30 min MVPA was significantly associated with a decreased risk of adverse cardiometabolic health (Table 3). Moreover, replacing MVPA with 30 min of sedentary behavior (OR: 1.14, 95%CI: 1.04–1.25), light intensity (OR: 1.15, 95%CI: 1.05–1.26), and lifestyle activities (OR: 1.17, 95%CI: 1.03–1.33) was significantly associated with an increased risk of adverse cardiometabolic health.

Table 4 shows the relationship between sedentary behavior and each type of physical activity with adverse cardiometabolic health (≥4 risk factors) in the single, partition, and IS models. We found that replacing sedentary behavior with MVPA was more closely associated with adverse cardiometabolic health (OR: 0.77, 95%CI: 0.68–0.87).

**Figure 1:**
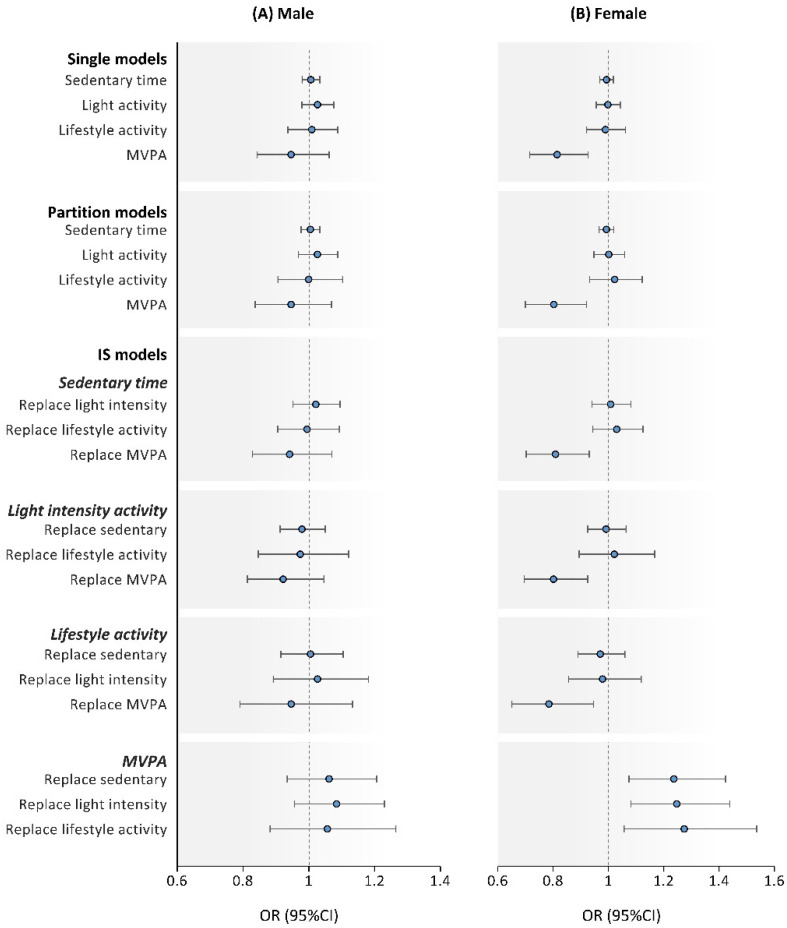
Single factor, partition, and isotemporal substitution models examining the association of sedentary behavior, light and lifestyle, and MVPA with adverse cardiometabolic health (≥3 risk factors) by male (**A**) and female (**B**). Note: Values were odds ratio (95%CI); each models including variables used models in Table 3 and Table 4. MVPA, moderate-to-vigorous physical activity; OR, odds ratio; CI, confidence interval; IS model, isotemporal substitution model.

**Figure 2:**
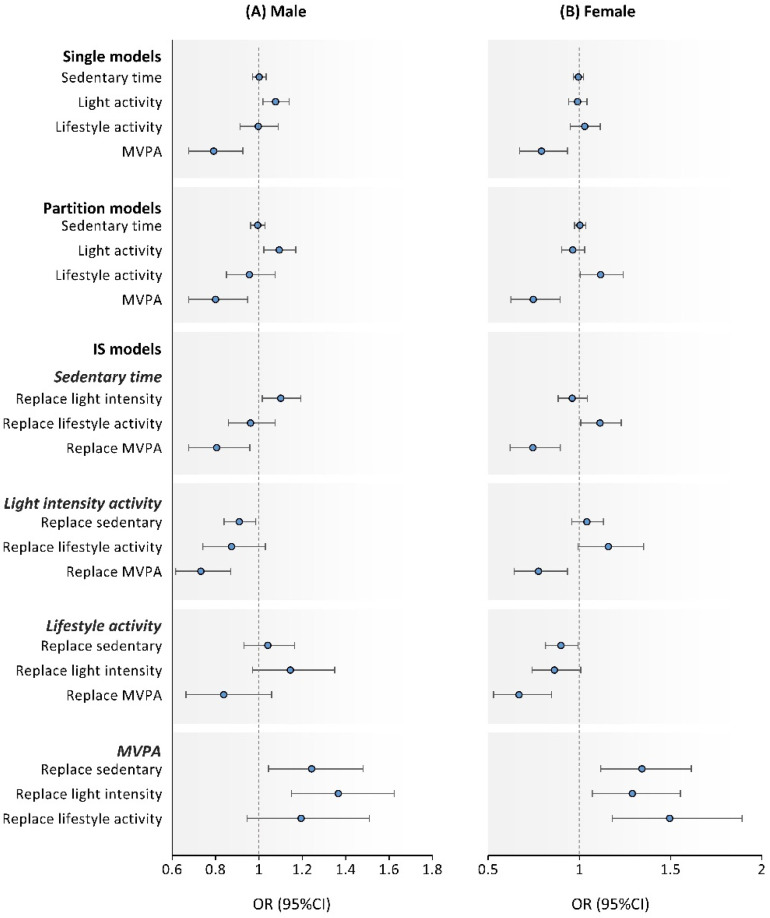
Single factor, partition, and isotemporal substitution models examining the association of sedentary behavior, light and lifestyle, and MVPA with adverse cardiometabolic health (≥4 risk factors) by male (**A**) and female (**B**). Note: Values were odds ratio (95%CI); each models including variables used models in Table 3 and Table 4. MVPA, moderate-to-vigorous physical activity; OR, odds ratio; CI, confidence interval; IS model, isotemporal substitution model.

We examined all analyses as a sensitivity analysis using models stratified by sex (men or women). Figure 1 shows a single, partition, and IS model based on sex. In men, despite showing a similar trend, the association of each type of activity and the risk of adverse cardiometabolic health was not significant in all models (Figure 1A). However, in women, a strong association with MVPA was found in the single, partition, and IS models (Figure 1B). For having ≥4 risk factors, these significant relationships were consistent in both men and women (Figure 2).

In addition, when we conducted analysis using stratified models by age group (<65 years or ≥65 years), the older group had significant associations with MVPA in all models; however, these associations were weaker in the younger group (Supplementary Figures S2 and S3).

## 4. Discussion

In this population-based cross-sectional study of Korean adults, we examined the association of accelerometer-measured sedentary behavior and type of physical activity with the risk of adverse cardiometabolic health and the potential effects of replacing sedentary behavior with other types of physical activity. This study is the first to describe the associations between accelerometer-measured sedentary behavior, physical activity, and adverse cardiometabolic risk factors among a large representative sample of Korean adults. We found that time spent in MVPA was significantly associated with a reduced risk of adverse cardiometabolic health. Moreover, IS models showed that replacing 30 min/day of sedentary behavior, light intensity, and lifestyle activities with an equivalent amount of MVPA was significantly associated with an elevated risk of adverse cardiometabolic health. In addition, the results showed that replacing MVPA with other types of activities had a harmful effect on cardiometabolic health. Our findings suggest that replacing sedentary behavior with MVPA may contribute to optimal cardiometabolic health among Korean adults.

In the partition models, we found a significant association between MVPA and the risk of adverse cardiometabolic health independently. However, sedentary behavior and other types of activities were not significant. The importance of physical activity for metabolic health in middle-aged and older adults has been confirmed in previous studies [12,27,29,30]. For example, a previous cross-sectional study reported that decreased objectively measured physical activity (light intensity and MVPA) was associated with increased metabolic risk factors and metabolic syndrome in a middle-aged men and women [15,29]. Other longitudinal and intervention studies have shown that MVPA is independently associated with an increased risk of cardiovascular disease, metabolic disorders (metabolic syndrome and type II diabetes), and mortality [8,14,18,31,32,33,34]. In addition, intervention investigations identified that increased MVPA was associated with improvement in metabolic disorders among overweight male employees [33]. The findings of the present study suggest that health promotion strategies can further emphasize the key factor of MVPA for cardiometabolic health in the Korean population.

Our study demonstrates that sedentary behavior can be replaced by other types of activities. To the best of our knowledge, this is the first investigation to explore the effects of replacing sedentary behavior with physical activity on the risk of cardiometabolic health using IS modeling in Korean adults. In the present study, replacing 30 min of sedentary behavior, light intensity, or lifestyle activity with an equal amount of MVPA was associated with a 12–15% reduction in the risk of adverse cardiometabolic health. On the other hand, replacing 30 min of MVPA with sedentary behavior or other types of activities was associated with a 14–17% elevation of cardiometabolic risk. Several previous studies on accelerometer-derived sedentary behavior and physical activity have described the potential effects of replacing sedentary behavior with an equal amount of light intensity activity or lifestyle activity (in other words, breaking sitting time or increasing standing time) to improve health [35,36,37,38,39]. Indeed, many epidemiological studies suggest that reduction in sedentary behavior may have a beneficial effect on health promotion independent of time spent on MVPA [12,15,20,32]. One accelerometer-based study reported that replacing sedentary behavior with physical activity was associated with cardiometabolic health in older adults [37]. Another study noted the effects of isotemporal substitution of sedentary behavior with light intensity or MVPA on cardiometabolic markers in male adolescents [38]. Moreover, most of these studies have described a consistent relationship that replacing MVPA with sedentary behavior or light intensity activity could have a harmful effect on cardiometabolic health.

In the sedentary population, substituting sedentary behavior into a lifestyle activity may be an effective approach rather than exercise training for health. However, contrary to the results of these studies, we could not find a significant association between replacing sedentary behavior and light intensity activity, and the risk of cardiometabolic health in all IS models. Several studies on the relationship between physical activity and metabolic disease suggest that the total volume of physical activity is an important factor and is more closely associated with metabolic biomarkers [40,41,42,43]. For example, Bassett et al. suggested that the total volume of physical activity might be a better metric than various types of activity, as it incorporates the full continuum of intensities [42]. Increasing the MVPA can effectively increase the total physical activity and energy expenditure.

Moreover, these results may suggest that the influence of high-intensity physical activity, such as MVPA, may be stronger than that of sedentary behavior on metabolic health. Traditional physical activity programs based on promoting MVPA have beneficial effects and have been recommended to improve metabolic health in the general population or in patients with metabolic disorders [33]. Moreover, recent studies have shown that participation in MVPA can reduce the risk of cardiovascular and metabolic diseases and mortality [18,31]. In addition, the 2020 WHO guidelines on physical activity and sedentary behavior recommend performing MVPA for at least 150 min/week [9]. Taken together, our results may support and expand on the results of previous studies on the association between MVPA and metabolic health.

We investigated sex differences in the associations between physical activity and the risk of adverse cardiometabolic health in the sensitivity analysis, although no significant interaction was observed. In a sex-stratified analysis among women, replacing sedentary behavior with MVPA resulted in a significantly lower risk of adverse cardiometabolic health. In men, despite showing similar results, these associations were weaker. Many previous studies have reported that sedentary behavior and physical activity are related to metabolic diseases and biomarkers in men and women. However, several studies have also shown sex differences in the association between physical activity patterns and cardiometabolic health [44,45,46]. The physiological differences by sex suggest that hormonal differences are linked to cardiometabolic risk factors [47].

The strength of this study is the use of a representative nationwide sample of Korean adults. We controlled for several sociodemographic variables (age, sex, education, and household income) and health-related behaviors (alcohol consumption and smoking status). In addition, we used standardized data and biological samples from a representative population of Korean adults, which makes it comparable to KNHANES and similar studies. Moreover, we used objectively measured activity parameters.

Our study has several limitations. First, the cross-sectional design of this study may limit the hypothesis of causality between sedentary behavior, physical activity, and cardiometabolic health. Therefore, future longitudinal or intervention studies should investigate the direction of the causality impact of the actual changing time of each behavior on cardiometabolic health. Second, although an accelerometer can objectively measure physical activity, there may be a limit to indicate a specific type of activity. In addition, further research is needed to confirm and extend our findings in other population. Finally, since the KNHANES did not measure hormone-related variables, we could not consider them in our models.

## 5. Conclusions

Our study found that replacing sedentary behavior, light intensity, and lifestyle activity with an equal time of MVPA was associated with a decreased risk of adverse cardiometabolic health in Korean adults by using IS modeling. Furthermore, replacing MVPA time with sedentary behaviors was associated with an increased risk of cardiometabolic health. Our findings suggest that reduction in sedentary behavior and increased MVPA may be useful for improving cardiometabolic health in Korean adults.

## Figures and Tables

**Table 1:** Participant characteristics.

	Overall (*n* = 2197)		Male (*n* = 826)		Female (*n* = 1371)		*p*-Value
Age [years] *^a^*	48.92	± 0.33	50.23	± 0.55	48.14	± 0.40	0.002
BMI [kg/m^2^]	23.80	± 0.07	24.47	± 0.11	23.39	± 0.09	<0.001
Waist circumference [cm]	80.95	± 0.21	85.68	± 0.30	78.10	± 0.24	<0.001
Systolic blood pressure [mmHg]	116.72	± 0.35	120.08	± 0.51	114.69	± 0.45	<0.001
Diastolic blood pressure [mmHg]	74.60	± 0.21	77.32	± 0.35	72.96	± 0.25	<0.001
Fasting blood glucose [mg/dL]	99.48	± 0.50	102.75	± 0.86	97.50	± 0.61	<0.001
Hemoglobin A1c [%]	5.69	± 0.02	5.74	± 0.03	5.66	± 0.02	0.020
HDL cholesterol [mg/dL]	51.83	± 0.26	47.48	± 0.39	54.45	± 0.33	<0.001
Triglyceride [mg/dL]	127.02	± 2.41	154.19	± 5.18	110.65	± 2.16	<0.001
Total cholesterol [mg/dL]	190.44	± 0.76	188.68	± 1.27	191.50	± 0.94	0.071
Cardiometabolic risk factors [numbers]	2.09	± 0.03	2.33	± 0.05	1.94	± 0.04	<0.001
Sedentary time (min/day)	465.45	± 3.47	465.93	± 5.79	465.16	± 4.33	0.914
Light intensity activity (min/day)	216.23	± 1.95	203.00	± 3.23	224.21	± 2.42	<0.001
Lifestyle activity (min/day)	79.42	± 1.18	80.35	± 2.00	78.86	± 1.45	0.537
MVPA (min/day)	32.63	± 0.73	38.51	± 1.31	29.09	± 0.84	<0.001
Wear time (min/day)	793.74	± 4.63	787.80	± 7.47	797.31	± 5.90	0.320
Education level [n (%)] *^b^*							
<High School	551	(25.08)	180	(21.79)	371	(27.06)	0.020
High School	807	(36.73)	312	(37.77)	495	(36.11)	
>High School	839	(38.19)	334	(40.44)	505	(36.83)	
Household income [n (%)]							
Q1	275	(12.52)	84	(10.17)	191	(13.93)	0.029
Q2	605	(27.54)	230	(27.85)	375	(27.35)	
Q3	674	(30.68)	249	(30.15)	425	(31.0)	
Q4	643	(29.27)	263	(31.84)	380	(27.72)	
Alcohol consumption [n (%)]							
Never	575	(26.17)	128	(15.5)	447	(32.6)	<0.001
Once a week	1209	(55.03)	422	(51.09)	787	(57.4)	
2–3 times/week	300	(13.65)	195	(23.61)	105	(7.66)	
≥4 times/week	113	(5.14)	81	(9.81)	32	(2.33)	
Smoking status [n (%)]							
Never	1547	(70.41)	276	(33.41)	1271	(92.71)	<0.001
Former	389	(17.71)	340	(41.16)	49	(3.57)	
Current	261	(11.88)	210	(25.42)	51	(3.72)	

Note: *^a^* Mean ± standard error (all such values). *^b^* Percentage (all such values). *p* values were calculated using *t*-test for continuous variables and chi-square test for categorical variables.

**Table 2:** Adverse cardiometabolic risk factors by sex.

	Overall (*n* = 2197)		Male (*n* = 826)		Female (*n* = 1371)		*p*-Value
Abdominal obesity [n (%)]	530	(24.12)	238	(28.81)	292	(21.30)	<0.001
High blood pressure [n (%)]	761	(34.64)	364	(44.07)	397	(28.96)	<0.001
High fasting glucose [n (%)]	712	(32.41)	342	(41.40)	370	(26.99)	<0.001
High hemoglobin A1c [n (%)]	178	(8.10)	78	(9.44)	100	(7.29)	0.074
High TG [n (%)]	576	(26.22)	311	(37.65)	265	(19.33)	0.004
Low HDL [n (%)]	747	(34.00)	199	(24.09)	548	(39.97)	<0.001
High total cholesterol [n (%)]	1078	(49.07)	389	(47.09)	689	(50.26)	0.151
Adverse cardiometabolic risk factors, ≥3 [n (%)]	817	(37.19)	372	(45.04)	445	(32.46)	<0.001
Adverse cardiometabolic risk factors, ≥4 [n (%)]	420	(19.12)	184	(22.28)	236	(17.21)	0.004

Note: *p* for difference was calculated using chi-square test for categorical variables.

**Table 3:** Single factor, partition, and isotemporal substitution models examining the association of sedentary behavior, light and lifestyle, and MVPA with adverse cardiometabolic health (≥3 risk factors).

	Sedentary Time		Light Intensity Activity		Lifestyle Activity		MVPA	
	OR	(95%CI)	OR	(95%CI)	OR	(95%CI)	OR	(95%CI)
Single model	0.99	(0.98, 1.01)	1.01	(0.98, 1.04)	1.01	(0.96, 1.06)	0.89	(0.81, 0.97)
Partition model	0.99	(0.98, 1.01)	1.00	(0.97, 1.04)	1.02	(0.96, 1.10)	0.87	(0.80, 0.96)
IS models								
Replace sedentary	*Dropped*		0.99	(0.94, 1.04)	0.97	(0.91, 1.03)	1.14	(1.04, 1.25)
Replace light intensity	1.01	(0.96, 1.06)	*Dropped*		0.98	(0.89, 1.08)	1.15	(1.05, 1.26)
Replace lifestyle activity	1.03	(0.97, 1.10)	1.02	(0.93, 1.12)	*Dropped*		1.17	(1.03, 1.33)
Replace MVPA	0.88	(0.80, 0.97)	0.87	(0.79, 0.96)	0.85	(0.75, 0.97)	*Dropped*	

Note: Single model: adjusted for age, sex, household income, education level, smoking status, alcohol consumption, and accelerometer wear time. Partition model: adjusted for single model covariates plus other types of activities without accelerometer wear time. IS model: dropped one activity from the full model including other types of activities and total ware time after adjusting for covariates. MVPA, moderate-to-vigorous physical activity; OR, odds ratio; CI, confidence interval; IS model, isotemporal substitution model.

**Table 4:** Single factor, partition, and isotemporal substitution models examining the association of sedentary behavior, light and lifestyle, and MVPA with adverse cardiometabolic health (≥4 risk factors).

	Sedentary Time		Light Intensity Activity		Lifestyle Activity		MVPA	
	OR	(95%CI)	OR	(95%CI)	OR	(95%CI)	OR	(95%CI)
Single model	0.99	(0.97, 1.01)	1.03	(0.99, 1.06)	1.02	(0.96, 1.08)	0.79	(0.70, 0.88)
Partition model	0.99	(0.97, 1.01)	1.02	(0.97, 1.07)	1.05	(0.97, 1.13)	0.76	(0.68, 0.86)
IS models								
Replace sedentary	*Dropped*		0.97	(0.92, 1.03)	0.95	(0.88, 1.02)	1.30	(1.14, 1.47)
Replace light intensity	1.03	(0.97, 1.09)	*Dropped*		0.97	(0.87, 1.09)	1.33	(1.18, 1.51)
Replace lifestyle activity	1.05	(0.98, 1.13)	1.03	(0.92, 1.15)	*Dropped*		1.37	(1.16, 1.61)
Replace MVPA	0.77	(0.68, 0.87)	0.75	(0.66, 0.85)	0.73	(0.62, 0.86)	*Dropped*	

Note: Single model: adjusted for age, sex, household income, education level, smoking status, alcohol consumption, and accelerometer wear time. Partition model: adjusted for single model covariates plus other types of activities without accelerometer wear time. IS model: dropped one activity from the full model including other types of activities and total ware time after adjusting for covariates. MVPA, moderate-to-vigorous physical activity; OR, odds ratio; CI, confidence interval; IS, isotemporal substitution model.

## Data Availability

Data used in this study are available in website of the Korea Centers for Disease Control and Prevention at https://knhanes.kdca.go.kr/knhanes/sub03/sub03_01.do.

## Supplementary Materials

The following are available online at https://www.mdpi.com/article/10.3390/ijerph182111102/s1, Figure S1: ActiGraph GT3X. Figure S2: Single factor, partition, and isotemporal substitution models examining the association of sedentary behavior, light and lifestyle, and MVPA with adverse cardiometabolic health (≥3 risk factors) by age group. Figure S3: Single factor, partition, and isotemporal substitution models examining the association of sedentary behavior, light and lifestyle, and MVPA with adverse cardiometabolic health (≥4 risk factors) by age group.
