# Supplementary material for: Isotemporal Substitution Analysis of Accelerometer-Derived Sedentary Behavior and Physical Activity on Cardiometabolic Health in Korean Adults: A Population-Based Cross-Sectional Study
Source: Int J Environ Res Public Health. 2021 Oct 22;18(21):11102. doi: 10.3390/ijerph182111102 (PMC8583396; doi:10.3390/ijerph182111102)
Supplement: Supplementary file 1 [file ijerph-18-11102-s001.zip › ijerph-1426540-supplementary.pdf]

Supplementary Materials

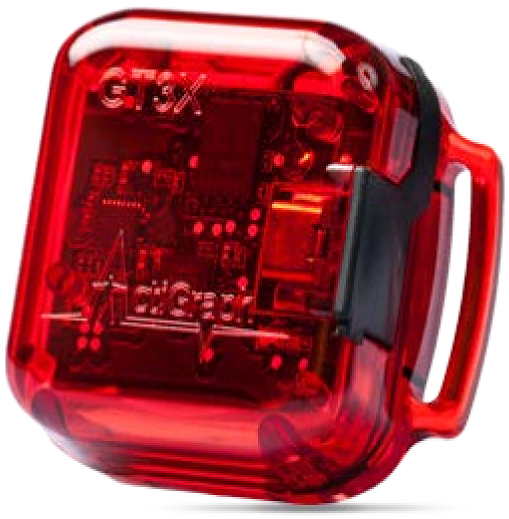

Figure S1. ActiGraph GT3X (LLC, Fort Walton Beach, FL, USA) https://actigraphcorp.com/support/activity-monitors/gt3x/

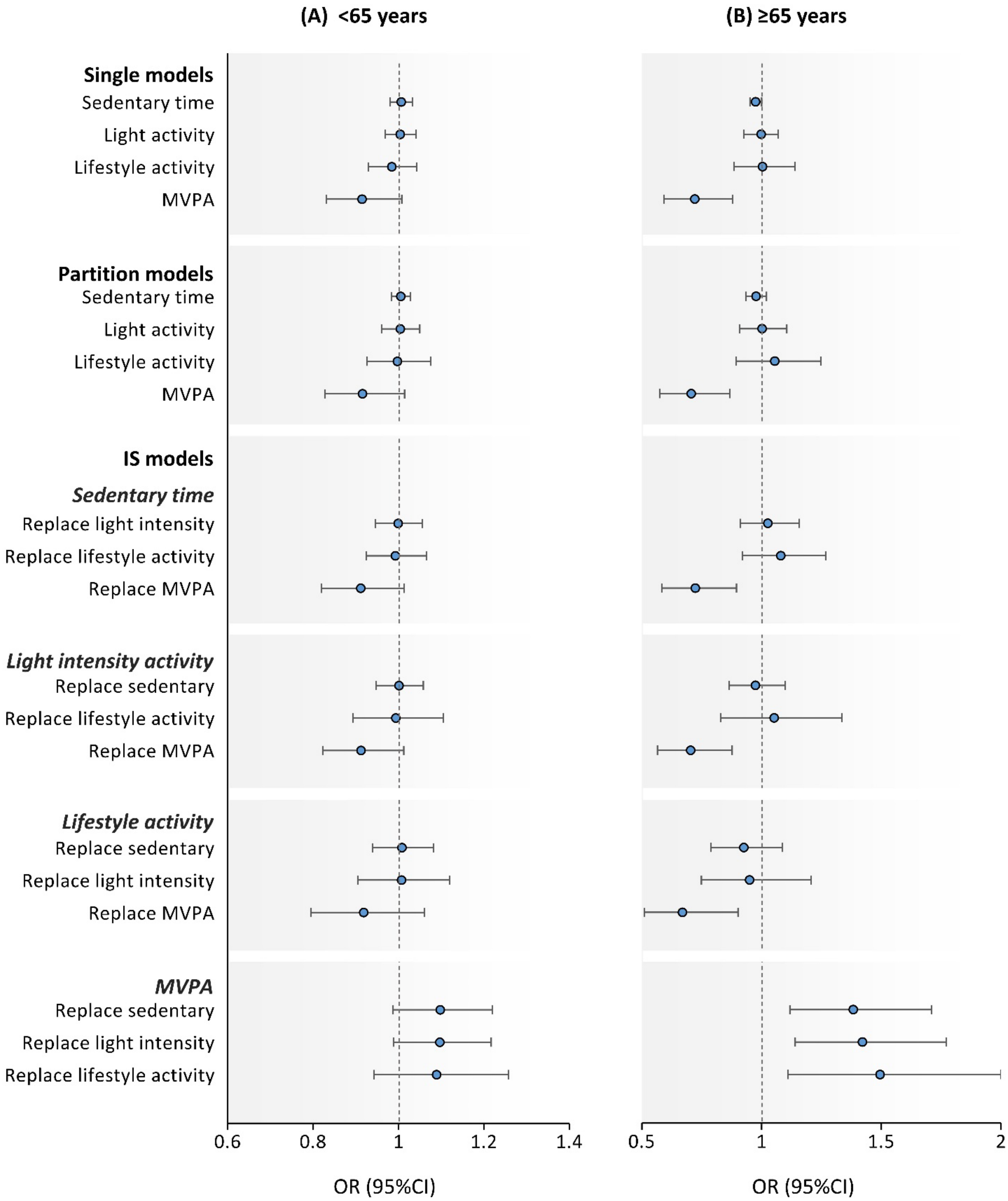

Figure S2. Single factor, partition, and isotemporal substitution models examining the association of sedentary behavior, light and lifestyle, and MVPA with adverse cardiometabolic health (≥3 risk factors) by age group

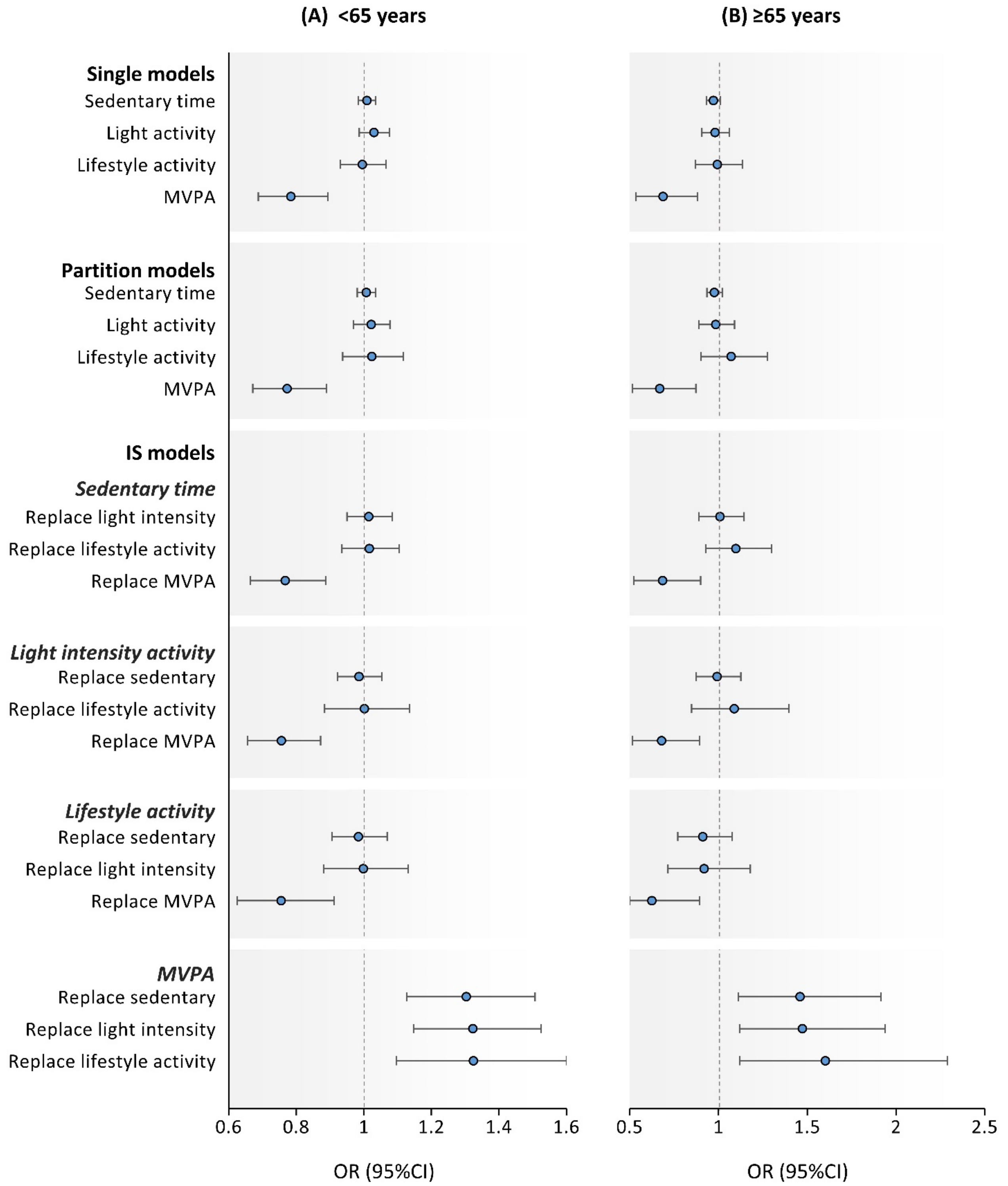

Figure S3. Single factor, partition, and isotemporal substitution models examining the association of sedentary behavior, light and lifestyle, and MVPA with adverse cardiometabolic health (≥4 risk factors) by age group
